# Biogenic selenium and tellurium nanoparticles synthesized by environmental microbial isolates efficaciously inhibit bacterial planktonic cultures and biofilms

**DOI:** 10.3389/fmicb.2015.00584

**Published:** 2015-06-16

**Authors:** Emanuele Zonaro, Silvia Lampis, Raymond J. Turner, S. Junaid S. Qazi, Giovanni Vallini

**Affiliations:** ^1^Department of Biotechnology, University of VeronaVerona, Italy; ^2^Biofilm Research Group, Department of Biological Sciences, University of CalgaryCalgary, AB, Canada

**Keywords:** antimicrobial activity, bacterial biosynthesis, biofilm growth mode, nanoparticles, selenium, tellurium

## Abstract

The present study deals with Se^0^- and Te^0^-based nanoparticles bio-synthesized by two selenite- and tellurite-reducing bacterial strains, namely *Stenotrophomonas maltophilia* SeITE02 and *Ochrobactrum* sp. MPV1, isolated from polluted sites. We evidenced that, by regulating culture conditions and exposure time to the selenite and tellurite oxyanions, differently sized zero-valent Se and Te nanoparticles were produced. The results revealed that these Se^0^ and Te^0^ nanoparticles possess antimicrobial and biofilm eradication activity against *Escherichia coli* JM109, *Pseudomonas aeruginosa* PAO1, and *Staphylococcus aureus* ATCC 25923. In particular, Se^0^ nanoparticles exhibited antimicrobial activity at quite low concentrations, below that of selenite. Toxic effects of both Se^0^ and Te^0^ nanoparticles can be related to the production of reactive oxygen species upon exposure of the bacterial cultures. Evidence so far achieved suggests that the antimicrobial activity seems to be strictly linked to the dimensions of the nanoparticles: indeed, the highest activity was shown by nanoparticles of smaller sizes. In particular, it is worth noting how the bacteria tested in biofilm mode responded to the treatment by Se^0^ and Te^0^ nanoparticles with a susceptibility similar to that observed in planktonic cultures. This suggests a possible exploitation of both Se^0^ and Te^0^ nanoparticles as efficacious antimicrobial agents with a remarkable biofilm eradication capacity.

## Introduction

In the last few decades, the emergence of bacterial resistance to antibiotics has become a common threat in both hospitals and community settings. As a consequence, the effectiveness of antibiotic treatment of bacterial infections has progressively decreased (Sievert et al., 2013). The problem is particularly relevant in the treatment of biofilm-associated infections, since bacteria grown in biofilm mode are more tolerant to conventional antibiotics and biocides compared to free swimming cells (Stewart and Costerton, 2001). Therefore, it is becoming increasingly imperative to develop and test new antimicrobial compounds capable of bactericidal activity even in biofilm growth mode toward multidrug resistant bacterial species.

In recent years, the utilization of metal ions and metal nanoparticles has emerged as an alternative to the use of organic compounds as antimicrobial agents (Lemire et al., 2013). Indeed, a widespread antimicrobial activity is often a common trait of nanomaterials, mainly due to the high surface to volume ratio of their constituent particles which results in a high reactivity. A vast literature exists dealing with the antimicrobial activity of silver nanoparticles (Sondi and Salopek-Sondi, 2004; Fabrega et al., 2009; Martinez-Gutierrez et al., 2013): however other metal or metalloid nanoparticles have shown so far bactericidal effects. For instance, the antimicrobial activity of both ZnO (Jones et al., 2008) and TiO_2_ (Tsuang et al., 2008) nanoparticles has been reported. In addition, selenium- (Tran and Webster, 2011) and tellurium-based (Mohanty et al., 2014) nanomaterials have revealed interesting antimicrobial potential against a broad range of pathogenic strains. This opens a new perspective for these nanoparticles in terms of coating agents in medical devices and health-related products to prevent bacterial infections (Roe et al., 2008). Moreover, they can find promising applications also in industrial settings as a potential tool to contrast biofouling (Zhang et al., 2012).

Nevertheless, currently the prominent drawback to the use of metal nanoparticles is the cost associated with their synthesis by means of traditional physico-chemical methods with production of toxic by-products (Narayanan and Sakthivel, 2010). For these reasons, nowadays a growing interest exists in developing new eco-friendly processes for the synthesis of potentially valuable nanoparticles. Among the possible alternatives, biogenesis represents an interesting option through the exploitation of the microbial ability to change the oxidation state of metal/metalloid salts with the final production of nanoparticles (Narayanan and Sakthivel, 2010). Such a green production of nanomaterials seems particularly suitable for the synthesis of stable monodisperse nanoparticles.

In this study we focused on the potential antimicrobial activity and biofilm eradication effect of zero-valent selenium and tellurium nanoparticles of biogenic origin on cultures of three biofilm-forming bacterial isolates, namely *Escherichia coli* JM109, *Pseudomonas aeruginosa* PAO1, and *Staphylococcus aureus* ATCC 25923. Experiments were carried out to test both the antimicrobial activity against either planktonic cells or cultures grown in biofilm mode and a possible involvement of reactive oxygen species as mechanism of toxicity of Se^0^ and Te^0^ nanoparticles toward the bacterial strains considered.

## Materials and methods

### Preparation of biogenic Se and Te nanoparticles

Two environmental isolates, *Stenotrophomonas maltophilia* SeITE02 (Di Gregorio et al., 2005) and *Ochrobactrum* sp. MPV1 (Santi, 2013) were used to produce respectively Se and Te NPs. Sterile Nutrient Broth supplemented with 0.5 mM Na_2_SeO_3_ or 0.3 mM K_2_TeO_3_ was inoculated with 1 mL of fresh inoculum. The culture was then incubated aerobically at 27°C. Bacterial cells and NPs were removed from culture medium after different time by centrifugation at 10,000 × g for 10 min. The pellets were washed twice with 0.9% NaCl solution, resuspended in Tris/HCl buffer (pH 8.2) and cells were then disrupted by ultrasonication at 100 W for 5 min. The suspension was then centrifuged at 10,000 × g for 30 min to separate disrupted cells (pellet) from NPs (supernatant). NPs were recovered after centrifugation at 40,000 × g for 30 min, washed twice and resuspended in deionized water.

### Dynamic light scattering (DLS) analysis

Measurements of Dynamic Light Scattering (DLS) from dispersed nanoparticles were made in Nanoscience lab at University of Calgary. Data were measured using a Zetasizer Nano-ZS by Malvern instrument with He—Ne laser at the wavelength of 633 nm and a power of 4.0 mW as a light source collecting data at a fixed scattering angle of 173°. 300 μL of the sample was applied to a quartz cell with a 10 mm path length and data collected at 25°C. From the autocorrelation function, the relaxation rate, Γ, is determined allowing for the translational diffusion coefficient, D, to be calculated using D = Γ/Q2, where Q in the magnitude of the scattering vector [Q = (4πn/λ) sin θ; n is the refractive index of the solution, λ the wavelength of the scattered light, and θ the scattering angle]. The viscosity of the water was taken as 8.9 × 10^−4^ Pa s and its refractive index as 1.33 at 25°C. The diffusion coefficients of the dispersed particles can be determined from the intensity of the autocorrelation function. Hydrodynamic diameter, Dh, can then be calculated from the diffusion coefficients, D, by using the Stokes-Einstein relation [Dh = (KBT)/3πηD; where KBT in thermal energy and η is the viscosity of the dispersion medium]. Our analysis used a cumulant fit to the correlation function and gives the averaged weighted diameter and a polydispersity index (PDI). A regularized fit to the DLS data gives more details on the size distribution of the dispersed nanoparticles. All the values were obtained using the software provided by the Malvern with the instrument.

### Scanning electron microscpy (SEM) analysis

The morphology and elemental composition of the purified Se- and Te-NPs were analyzed through scanning electron microscopy (SEM). Nanoparticles were fixed, dehydrated with increasing ethanol concentrations and dried through the critical point method by using liquid CO_2_. They were mounted on metallic specimens stubs and then directly observed through XL30 ESEM (FEI-Philips) equipped with an EDAX micro-analytical system.

### Evaluation of MIC (minimun inhibitory concentration)

Minimum inhibitory concentration of Se and Te-NPs_6H_ was evaluated against the following five different strains: *E. coli* JM109, *E. coli* ATCC 25922, *P. aeruginosa* PAO1, *P. aeruginosa* ATCC 27853, and *S. aureus* ATCC 25923. Briefly, in a 96-well microtitre plate, wells were inoculated with 75 μL of 1/15 dilution of a 1.0 McFarland standard, LB broth and increasing concentrations of NPs (7.8125, 15.6250, 31.25, 62.5, 125, 250, 500, 1000, 2000, 4000, 8000 mg/L). After 24 h of incubation at 37°C, cultures were serially diluted and spot plated onto Nutrient Broth agar plates. After 48 h of incubation at 37°C, viability was quantified by counting colonies. The MIC values were recorded as the lowest concentrations of NPs that completely inhibited bacterial growth of the different strains tested.

### Antimicrobial activity evaluation

The antimicrobial activity of the different nanoparticles produced was evaluated against three reference strains: *E. coli* JM109, *P. aeruginosa* PAO1, and *S. aureus* ATCC 25923. A modification of the MBEC™ protocol (Harrison et al., 2010) was utilized. In a 96-well microtitre plate, all wells were inoculated with 75 μL of 1/15 dilution of a 1.0 McFarland standard and 75 μL of LB broth. The MBEC™ lid was then attached and cultures were incubated 37°C, 95% humidity, shaken at 125 rpm. After 48 h of growth to establish the biofilms, the MBEC™ lid was moved and exposed to another 96-well microtitre plate, containing an increasing gradient of nanoparticles concentrations. The viability of the bacterial cultures was evaluated using the established MBEC™ recovery protocol (Harrison et al., 2010) after 4 h of exposure, to test the ability of the nanoparticles to inhibit biofilm formation, and 24 h of exposure after 24 h of growth, to evaluate the biofilm eradication capability.

Bioflms were recovered by rinsing MBEC lid twice in 200 μL saline for 1 min and sonicating for 10 min at 60 Hz in a 96-well microtitre plate containing 200 μL Nutrient Broth, 0.1% Tween-20 and 1/50 diluted universal neutralizer. The planktonic cultures were also recovered by adding 10 μL of a 1/10 dilution of universal neutralizer to 40 μL of cultures. Both biofilm and planktonic cultures were serially diluted and spot plated onto Nutrient Broth agar plates. After 48 h of incubation at 37°C, viability was quantified by counting colonies.

The effective concentration causing 50% growth inhibition (EC_50_) was calculated with GraphPad Prism 6.01 software. All tests were carried out in triplicate (*n* = 3) and the results were averaged.

### Confocal laser scanning microscopy (CSLM) analysis

Biofilms were visualized by CLSM using both the fluorescent stain Acridine Orange (AO), the LIVE/DEAD® BacLight™ Bacterial Viability Kits and a Leica DM IRE2 microscope with a 64× water immersion objective. 3D images were generated using Imaris x64 Image Processing Software (Bitplane Scientific Software, South Windsor, CT, USA).

### Quantification of reactive oxygen species (ROS)

The ROS formed after the exposition of bacterial culture to SeNPs or TeNPs were quantified using 2′-7′-dichlorofluoresceindiacetate (DCFDA) (Wang and Joseph, 1999). The concentration of nanoparticles used was 30 mg/L. DCFDA interaction with NPs alone was tested. Cultures were incubated at 37°C for 3 h and then centrifuged at 4°C for 30 min at 300 × g. DCFDA was then added to the supernatant at a concentration of 100 μM. After 1 h, ROS were quantified at 485/20 nm of fluorescence excitation wavelength and 528/20 nm of emission wavelength using Gemini EM Fluorescence Microplate Reader (MolecularDevices, U.S.A.). Differences between the ROS production induced by the different nanoparticles were determined using One-Way analysis of variance (ANOVA). The level of significance was set at *P* < 0.05. All tests were carried out in triplicate (*n* = 3) and the results were averaged.

## Results

### Biogenesis and characterization of Se and Te nanoparticles

Different types of NPs were produced using *Stenotrophomonas maltophilia* SeITE02 and *Ochrobactrum* sp. MPV1. The two strains had previously shown the ability to reduce respectively, selenite (Di Gregorio et al., 2005) and tellurite to the elemental forms. NPs were produced after 6 h of bacteria incubation with the oxyanions (SeNPs_6H_ and TeNPs_6H_), 24 h of incubation (SeNPs_24H_ and TeNPs_24H_), and 48 h (SeNPs_48H_ and TeNPs_48H_).

**Figure 1:**
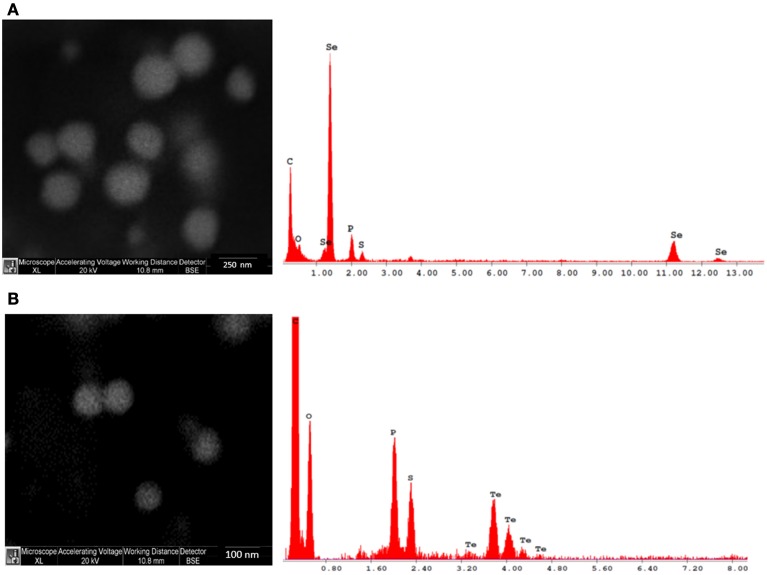
**Scanning electron microscopy (SEM) and energy dispersive X-ray (EDX) images of Se (A) and Te (B) NPs synthesized after 48 h of incubation**.

SEM observations indicate that both Se and Te are well dispersed and of spherical shape (Figure 1).

EDX microanalysis of the purified SeNPs and TeNPs exhibited respectively the characteristic Se absorption peaks at 1.37, 11.22, and 12.49 keV and the Te absorption peak at 3.769 keV. Moreover, EDX analysis revealed also the presence of the absorption peaks of C, O, P, and S, indicating the possible association of the purified Se and Te NPs with organic compounds.

**Figure 2:**
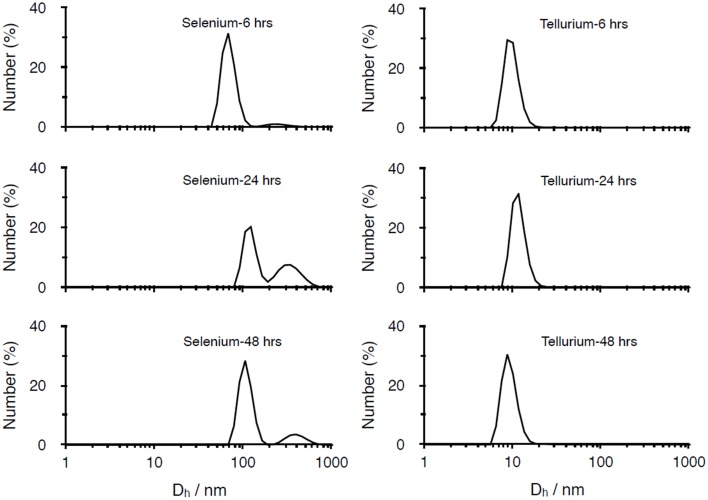
**DLS analysis of Se and Te-based nanoparticles produced after 6, 24, 48 h of incubation**.

The different kinds of NPs produced were also characterized by using dynamic light scattering (DLS) technique (Figure 2). For what concerns SeNPs, the diameters of the NPs increase with the incubation times. After 6 h of incubation, the average hydrodynamic diameter of the NPs, calculated through cumulant fit, is 221.1 nm, with a polydispersity index (PDI) of 0.24. As the incubation time increases to 24 and 48 h, also the diameter of the NPs grows to values of 345.2 nm (PDI = 0.21) and 357.1 nm (PDI of 0.24), respectively. On the other hand, the diameter of TeNPs remains almost stable as the incubation time increases, with values of 78.5 nm (PDI = 0.50) after 6 h of incubation, 76.2 nm (PDI = 0.49) after 24 h and 76.1 nm (PDI = 0.50) after 48 h.

### Evaluation of MIC values

MIC values obtained with Se and Te-NPs_6H_ for *E. coli* JM109, *E. coli* ATCC 25922, *P. aeruginosa* PAO1, *P. aeruginosa* ATCC 27853 and *S. aureus* ATCC 25923 are shown in Table 1. The values with SeNPs_24H_ were 125 mg/L for *E.coli* JM109 and *E. coli* ATCC 25922 while 250 mg/L for *P. aeruginosa* PAO1, *P. aeruginosa* ATCC 27853 and *S. aureus* ATCC 25923. On the other hand, the value with TeNPs_6H_ was 500 mg/L for all strains tested, except for the test with *S. aureus* ATCC 25923 in which a MIC of 1000 mg/L was registered.

**Table 1:** **MIC (minimun inhibitory concentration) of Se and Te-NPs_6H_against** ***E. coli*** **JM109,** ***E. coli*** **ATCC 25922,** ***P. aeruginosa*** **PAO1,** ***P. aeruginosa*** **ATCC 27853, and** ***S. aureus*** **ATCC 25923**.

**Strains**	**MIC SeNPs_6H_ (mg/L)**	**MIC TeNPs_6H_ (mg/L)**
*E. coli* JM109	125	500
*E. coli* ATCC 25922	125	500
*P. aeruginosa* PAO1	250	500
*P. aeruginosa* ATCC 27853	250	500
*S. aureus* ATCC 25923	250	1000

### Evaluation of antimicrobial and antibiofilm activity of nanoparticles

The susceptibility of *E. coli* JM109, *P. aeruginosa* PAO1 and *S. aureus* ATCC 25923 biofilm and planktonic population to the different biogenic NPs was evaluated. Analyses were performed on only one strain for each bacterial species considered. The antimicrobial activities of NPs were compared with the toxic effect of the correspondent metal salt. Viable cell counts were determined for increasing NPs concentrations after 4 h of growth with NPs (to test their ability to inhibit biofilm formation) and 24 h of nanoparticles exposure of pre-established biofilms (to verify their biofilm-eradication activity).

**Figure 3:**
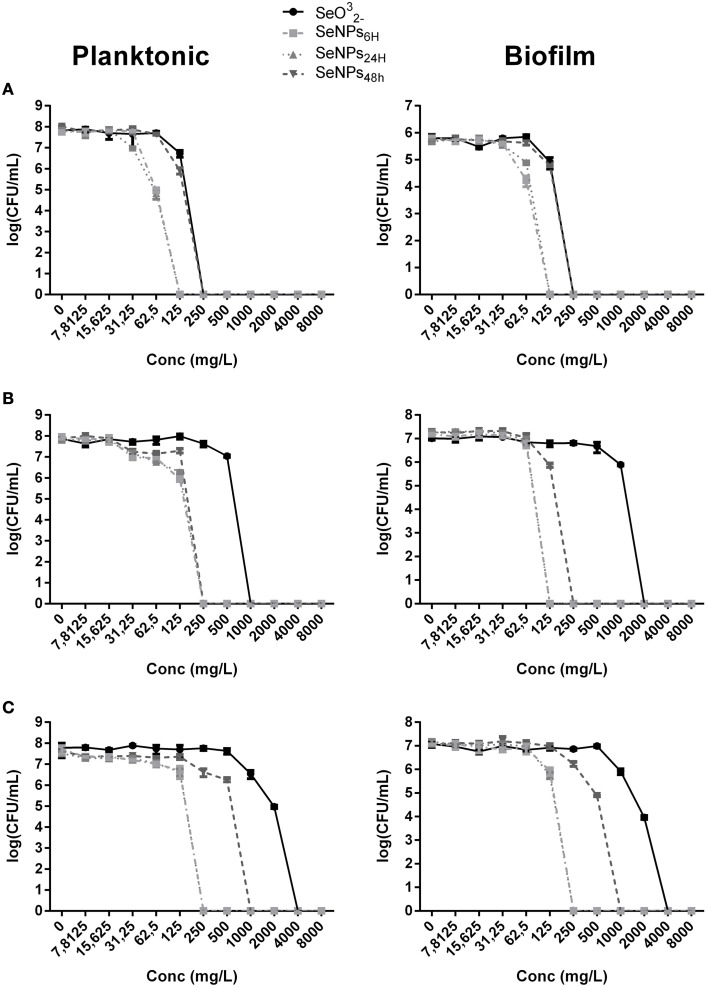
**MBEC assays performed on the planktonic and biofilm population of** ***E. coli*** **JM109 (A),** ***P. aeruginosa*** **PAO1 (B), and** ***S. aureus*** **ATCC 25923 (C) exposed to increasing concentrations of SeNPs (*****n*** **= 3)**.

In Figure 3, the effect of 4 h exposure to SeNPs is summarized. In each one of the experiments, SeNPs exhibited the ability to kill 100% of both planktonic and biofilm populations. Moreover, SeNPs_6H_ and SeNPs_24H_ showed generally a higher antimicrobial and antibiofilm activity when compared with SeNPs_48H_ and selenite. For *E. coli* JM109 planktonic culture, we detected a toxic effect of SeNPs_6H_ and SeNPs_24H_ already at a concentration of 62.5 mg/L with a 3 Log reduction. We achieve 100% kill of the planktonic culture at a SeNPs concentration of 125 mg/L. A similar pattern was observed for the other two strains tested. For both *P. aeruginosa* PAO1 and *S. aureus* ATCC 25923, the concentration of SeNPs_6H_ and SeNPs_24H_ required to kill 100% of the planktonic population was 250 mg/L. When we analyze the toxicity of SeNPs toward biofilm culture, we achieved the 100% kill of the culture at the same NPs concentration required to kill planktonic population, for both strains.

**Figure 4:**
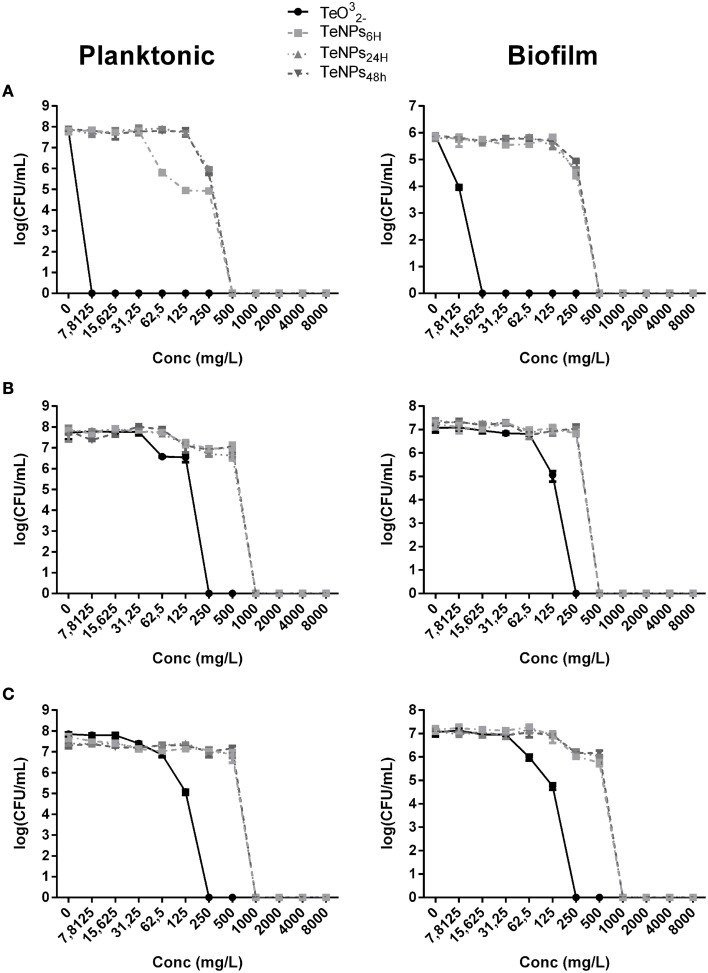
**MBEC assays performed on the planktonic and biofilm population of** ***E. coli*** **JM109 (A),** ***P. aeruginosa*** **PAO1 (B), and** ***S. aureus*** **ATCC 25923 (C) exposed to increasing concentrations of TeNPs (*****n*** **= 3)**.

We can observe the toxic effect of 4 h exposure to TeNPs toward planktonic and biofilm cultures in Figure 4. In this case, tellurite exhibited the highest toxicity and different sized TeNPs showed similar toxicity between each other for the strain tested. For *E. coli* JM109 and *P. aeruginosa* PAO1, a concentration of 500 mg/L of TeNPs was required to kill 100% of planktonic cultures, while *S. aureus* ATCC 25923 culture was completely eradicated at a concentration of 1000 mg/L. Biofilms were eradicated at the same TeNP concentrations that killed planktonic cultures, showing a similar resistance to TeNPs.

**Figure 5:**
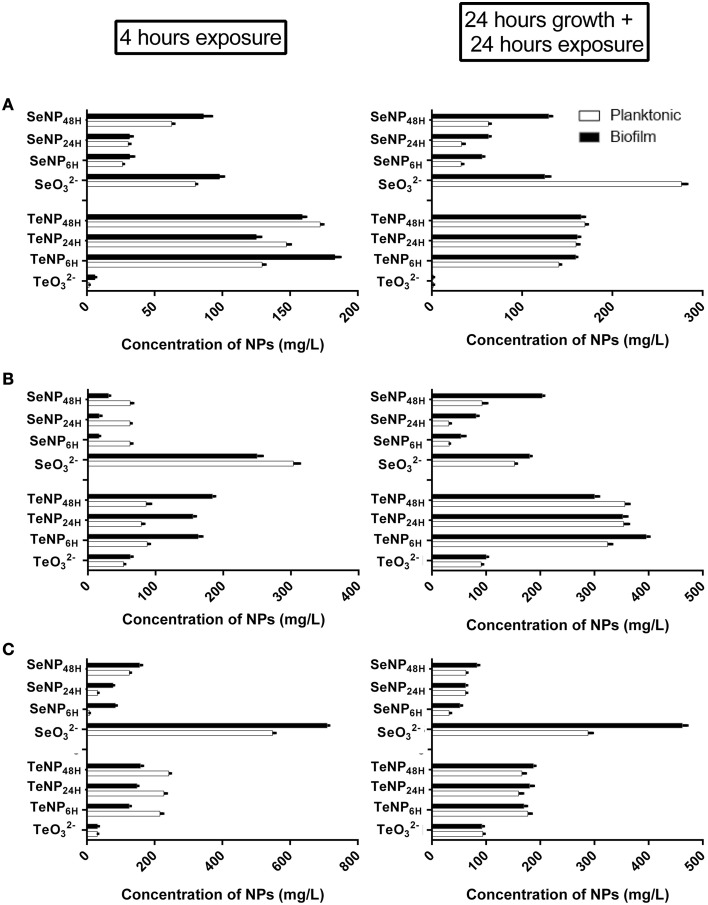
**EC_50_ of SeNPs and TeNPs for planktonic and biofilm cultures of** ***E. coli*** **JM109 (A),** ***P. aeruginosa*** **PAO1 (B), and** ***S. aureus*** **ATCC 25923 (C) after 4 h of exposure and 24 h of growth + 24 h of exposure**.

EC_50_ analysis (Figure 5) confirmed the higher toxicity of SeNPs_6H_ and SeNPs_24H_ toward both planktonic and biofilm populations. For instance, EC_50_ of SeNPs_6H_ and SeNPs_24H_ for planktonic cultures of *E. coli* was respectively 26.32 mg/L and 30.51 mg/L, while EC_50_ for SeNPs_48H_ was two time higher, 62.66 mg/L. On the other hand, EC_50_ are similar between the different TeNPs tested, showing no influence of incubation time for TeNPs antimicrobial activity. Even in this case, for both SeNPs and TeNPs, biofilm cultures didn't exhibit a higher tolerance toward the antimicrobial action of the NPs when compare to planktonic populations.

### CLSM analysis of biofilm cultures exposed to nanoparticles

Another technique to characterize the antimicrobial effect of nanoparticles against biofilm culture is Confocal Laser Scanning Microscopy with LIVE/DEAD® *Bac*Light™ Bacteria Viability Kit (Life Technology). This staining uses two different dyes: Syto9, a nucleic acid intercalator which fluoresce in green in living cells, and propidium iodide, which fluoresce in red after binding DNA or RNA of dead cells. By using this two dyes in conjunction, we obtain biofilm images in which viable cells are stained in green and dead cells in red.

After 24 h of growth in MBEC device, the strains formed a surface-adherent layer of bacterial cells with microcolonies of about 10–15 μm in height.

**Figure 6:**
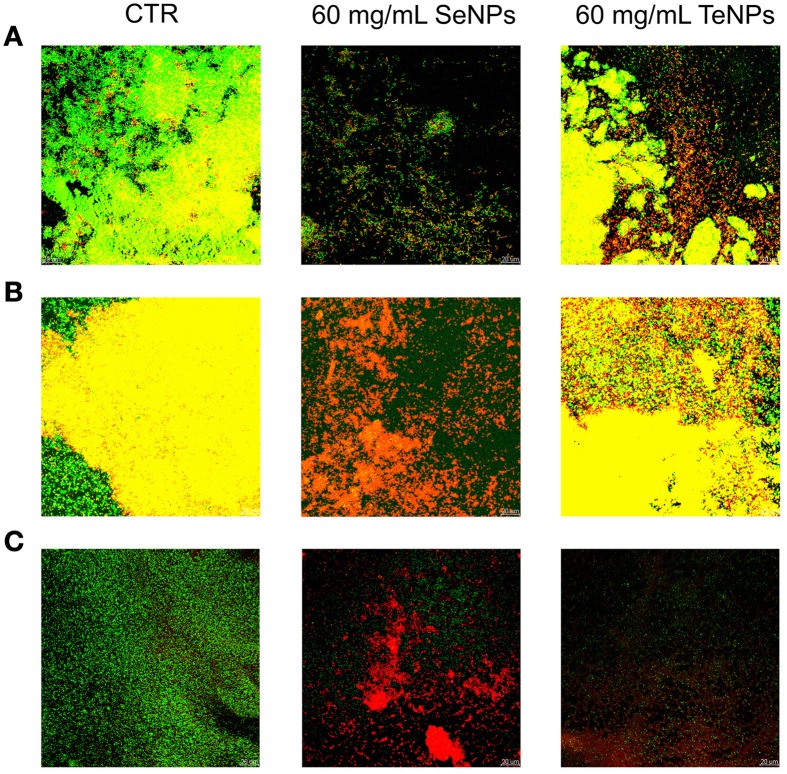
**Confocal microscopy performed on biofilms of** ***E. coli*** **(A),** ***P. aeruginosa*** **(B), and** ***S. aureus*** **(C) exposed to 60 mg/L of nanoparticles**.

As we can see from Figure 6 we can detect an effect of the NPs on biofilm structure for each one of the bacteria analyzed. At a concentration of 60 mg/L, SeNPs completely eradicated the biofilm structure of *E. coli*. At the same concentration, SeNPs killed most part of biofilms cells of both *P. aeruginosa* and *S. aureus*, in contrast to growth control, formed for the most part of viable cells. On the other hand, at the same concentration of 60 mg/L, TeNPs exhibited a lower toxicity effect toward biofilms structure: large portions of the biofilms are still composed by alive cells, in particular on the interior region of the structure. Nevertheless, biofilms in presence of TeNPs had a decreased thickness and less pronounced structural features.

These results confirm that biogenic NPs have antibiofilm activity against the three strains tested, as we have already seen with the viable cells count.

### Detection of reactive oxygen species (ROS)

In order to explore the mechanism of NPs toxicity, the production of reactive oxygen species (ROS) was evaluated in response to the NPs exposure. *E. coli* JM109, *P. aeruginosa* PAO1 and *S. aureus* ATCC 25923 were exposed for 3 h to the same concentration of NPs (30 mg/L) and reactive oxygen species (ROS) were quantified both in planktonic and biofilm cultures. Controls with different concentrations of both Se- and Te-NPS were performed and we didn't detect interactions between nanoparticles and DCDFA (Figure S1).

**Figure 7:**
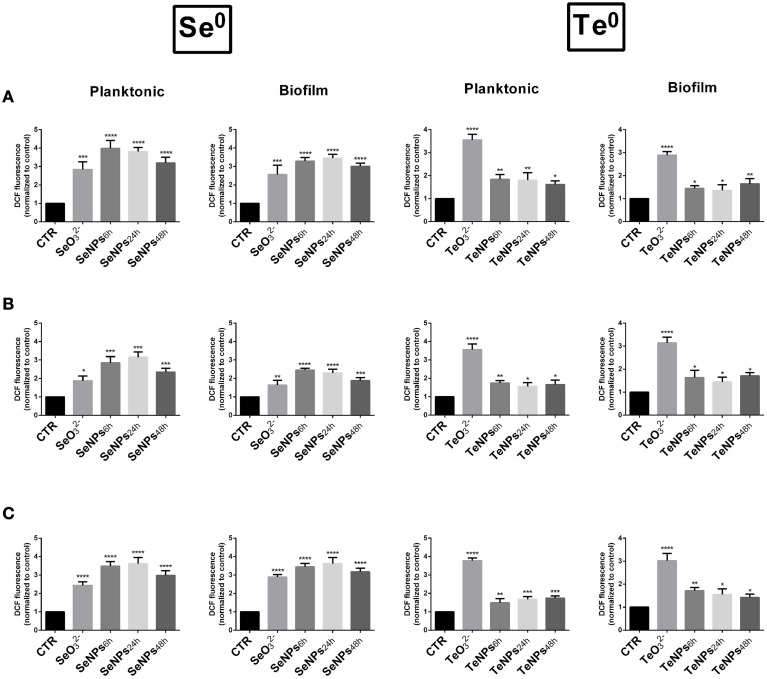
**Formation of ROS in** ***E. coli*** **(A),** ***P. aeruginosa*** **(B), and** ***S. aureus*** **(C) cells exposed to 30 mg/L of SeNPs and TeNPs. (*****n*** **= 3;** ***P*** **< 0.05)**.

In planktonic cultures, SeNPs induced a higher amount of ROS than selenite for each one of the strains tested (*P* < 0.05). Among the different SeNPs, there isn't a significant difference among nanoparticles produced after different incubation times. ROS production induced by TeNPs was, on the other hand, lower than that obtained after exposure to tellurite (Figure 7).

The same behavior was detected in biofilm cultures, with a similar amount of ROS generated.

## Discussion

It is already well known that both metals and metalloids in their different ionic forms can exhibit a strong antimicrobial effect against microbial cells (Lemire et al., 2013). In recent years, however, a variety of nanostructured metals has evidenced very promising antibacterial properties. The uses of these metal nanoparticles have potential advantages over conventional antimicrobial agents due to their high surface to volume ratios that allow a higher area of interactions with biological systems. Nanomaterials also have unique physical and chemical properties that may lead to greater efficacy as a biocide. The present work shows that both biogenic Se^0^ and Te^0^ nanoparticles exhibited antimicrobial and anti-biofilm activity against *E. coli*, *P. aeruginosa* and *S. aureus*.

We report in the present study that biogenic SeNPs synthesized by the strain *Stenotrophomonas maltophilia* SeITE02 show characteristics analogous to those of previously described selenium nanoparticles of microbial origin. For instance, Lampis and coworkers reported that SeNPs from *Bacillus mycoides* SeITE01 owned spherical morphology and dimensions similar to the biogenic nanoparticles here discussed (Lampis et al., 2014). Indeed, a spherical morphology and dimensions between 100 and 400 nm are also typical of other biogenic SeNPs characterized elsewhere (Husen and Siddiqi, 2014).

On the other hand, there are far fewer descriptions on biogenic tellurium nanoparticles in literature. Zare and coworkers reported the purification of rod-shaped TeNPs from *Bacillus* sp. BZ (Zare et al., 2012). However, the nanoparticles produced by this strain evidenced significant differences with reference to either morphology or sizes compared to TeNPs analyzed in the present study.

In our investigation, TeNPs generated by *Ochrobactrum* sp. MPV1 showed a lower antimicrobial activity than tellurite while SeNPs exerted a higher biocidal effect on microbial growth than selenite. This latter finding is surprising, since SeNPs have previously been reported to exhibit a lower toxicity with respect to the corresponding oxyanion, either in *in vivo* or *in vitro* tests (Shakibaie et al., 2013). Moreover, we demonstrated here that the antimicrobial activity of SeNPs shows a size-dependent response as already suggested by Lu et al. (2013). Smallest SeNPs_6H_, among the different dimensional classes of nanoparticles obtained in this study, exhibited the strongest antimicrobial activity with an EC_50_ of 26.32 mg/L for *E. coli*, 7.59 mg/L for *S. aureus* and 62.37 mg/L for *P. aeruginosa*, respectively, and the ability to completely inhibit biofilm formation at a concentration of 125 mg/L for *E. coli* and *P. aeruginosa* and 250 mg/L for *S. aureus*. As nanoparticles decrease in size, their surface to volume ratio increase, confirming smaller is better for improving the biological reactivity. Nevertheless, size is not the sole parameter influencing antimicrobial properties of the nanoparticles: other important features are both the elemental composition and the shape (Pal et al., 2007). In this respect, for the antimicrobial activity of TeNPs, we did not detect a significant difference between NPs produced at different incubation times (leading to different sizes). The EC_50_ values of differently sized TeNPs were 160 mg/L for *E. coli*, 150 mg/L for *S. aureus*, and 175 mg/L for *P. aeruginosa*.

Antimicrobial activity of various biogenic SeNPs and TeNPs has been explored before, however, different methodologies were used to evaluate the bactericidal activity. Although particles and protocols are different, the resistance levels reported in literature are similar to the concentrations found in this work. In particular, SeNPs produced by *Bacillus* sp. MSh-1 resulted in the inhibition of biofilm formation by *S. aureus* ATCC 25923, *P. aeruginosa*, and *P. mirabilis* already at a concentration of 16 mg/L, with a decrease of biofilm formation of 58% for *S. aureus*, 65.7% for *P. aeruginosa* and 46.6% for *P. mirabilis* (Shakibaie et al., 2015). Chemically synthesized SeNPs have also been reported as capable of antimicrobial activity. Tran and Webster studied the effect of SeNPs on *S. aureus* ATCC 25923. Their observations indicate that SeNPs cause a 3 log inhibition of *S. aureus* growth at concentrations between 7.8 and 31 mg/L after 3 h of exposure (Tran and Webster, 2011). Moreover, Chudobova et al. reported their chemically synthesized SeNPs with a higher antimicrobial activity than AgNPs, inducing a complete growth inhibition of *S. aureus* NCTC 8511 at a concentration of 23.7 mg/L (Chudobova et al., 2014).

Biogenic TeNPs have also been studied for their potential bactericidal activity. Zare et al. (2012) demonstrated antibacterial activity of TeNPs against different clinical isolates (*S. aureus*, *P. aeruginosa*, *S. typhi* and *K. pneumonia*), with an MBC (minimum bactericidal concentration) between 125 and 500 mg/L (Zare et al., 2012). Nevertheless, by comparing the antimicrobial activity of SeNPs and TeNPs with silver nanoparticles (AgNPs), these latter generally show antimicrobial activity at lower concentrations than both SeNPs and TeNPs. In fact, AgNPs completely inhibited biofilm formation in *E. coli* AB1157 and *P. aeruginosa* PAO1 at a concentration of 5 and 10 mg/L, respectively. However, the concentration required to eradicate an already formed biofilm structure is over 150 mg/L (Radzig et al., 2013), similar to the values reported in the present study for biogenic SeNPs and TeNPs.

An unexpected finding in the present investigation is that both the biogenic SeNPs and TeNPs exhibited equivalent antimicrobial efficacy toward the bacterial cells grown either in plankonic form or in biofilm mode. This evidence is particularly significant since bacteria grown in biofilm form are reported to be more tolerant to antimicrobial agents, in particular to traditional antibiotics (Hall-Stoodley et al., 2004).

On the basis of both the evaluation of antimicrobial activity and CLSM observations, it appears evident that SeNPs and TeNPs possess bactericidal properties. Anyway, the mechanisms responsible for the antimicrobial activity are not yet completely understood. One of the possible modes of action that has been proposed to explain the toxicity of other nanomaterials (AgNPs and ZnNPs) is the production of reactive oxygen species (ROS) (Manke et al., 2013). Reports already exist in the literature that attribute the antibacterial effects of different selenium compounds to the formation of free radicals (Tran et al., 2009). Moreover, selenium and tellurium oxyanions have also been found to trigger the generation of ROS, with both elements capable of reacting with intracellular thiols and forming intermediates that cause oxidative stress as a consequence of the formation of superoxide radicals (Zannoni et al., 2008). In the present study, after TeNPs exposition, the amount of ROS induced is lower than that recorded after tellurite exposure. On the other hand, we observed that SeNPs induce a higher production of ROS compared to selenite. However, we did not observe induction of ROS formation by SeNPs in a size-dependent manner. In fact, the amounts of ROS generated by different dimensional types of SeNPs were very similar, even if the toxicity effects they exert to the microbial populations increase with the lowest dimensions. Thus, even if reactive oxygen species are involved in the toxicity of NPs, there must be other mechanisms responsible for the antimicrobial activity of these nanostructured metals. For instance, it seems that nanoparticles can contribute to functional damages of cell membrane or wall by disrupting the integrity of these important envelopes (Pi et al., 2013). Some other mechanism related to the surface features of the nanoparticles may be involved however in conferring toxicity to NPs (Bao et al., 2015).

In conclusion, the study presented here concerns the analysis of the antibacterial properties of elemental selenium and tellurium nanoparticles of biogenic origin against reference strains of biofilm-forming bacteria tested either in planktonic cultures or as biofilms. Both Se^0^ and Te^0^ nanoparticles have demonstrated to efficaciously inhibit planktonic and biofilm growth of *E. coli* JM109, *P. aeruginosa* PAO1, and *S. aureus* ATCC 25923. It is worth noting that these nanoparticles evidenced the ability to hinder biofilm formation and, likewise, to completely eradicate already established biofilms. In particular, antimicrobial activity of SeNPs is size dependent. Another element of absolute novelty is that Se^0^ nanoparticles showed a higher antimicrobial activity than the free oxyanion selenite (SeO^2−^_3_) well recognized to be quite toxic to biota. Indeed, previous works on antimicrobial activity of metal nanoparticles reported that anionic forms are usually more toxic to bacteria than the corresponding nanoparticles. The evidence that Se^0^ and Te^0^ nanoparticles—obtained through the reduction of selenite and tellurite by *Stenotrophomonas maltophilia* SeITE02 and *Ochrobactrum* sp. MPV1, respectively—are capable of inducing the formation of ROS as possible cause of microbial growth inhibition deserves also particular attention. Recently, the activity of a different form of biogenic Se^0^ nanoparticles was explored against biofilm cultures (Shakibaie et al., 2015).

The evidence gained in this study and the results of other recent investigations strengthen the perspective of a possible use of SeNPs and TeNPs as efficacious antimicrobial agents.

### Conflict of interest statement

The authors declare that the research was conducted in the absence of any commercial or financial relationships that could be construed as a potential conflict of interest.
