# Supplementary material for: Biogenic selenium and tellurium nanoparticles synthesized by environmental microbial isolates efficaciously inhibit bacterial planktonic cultures and biofilms
Source: Front Microbiol. 2015 Jun 16;6:584. doi: 10.3389/fmicb.2015.00584 (PMC4468835; doi:10.3389/fmicb.2015.00584)
Supplement: Supplementary file 1 [file Image1.PDF]

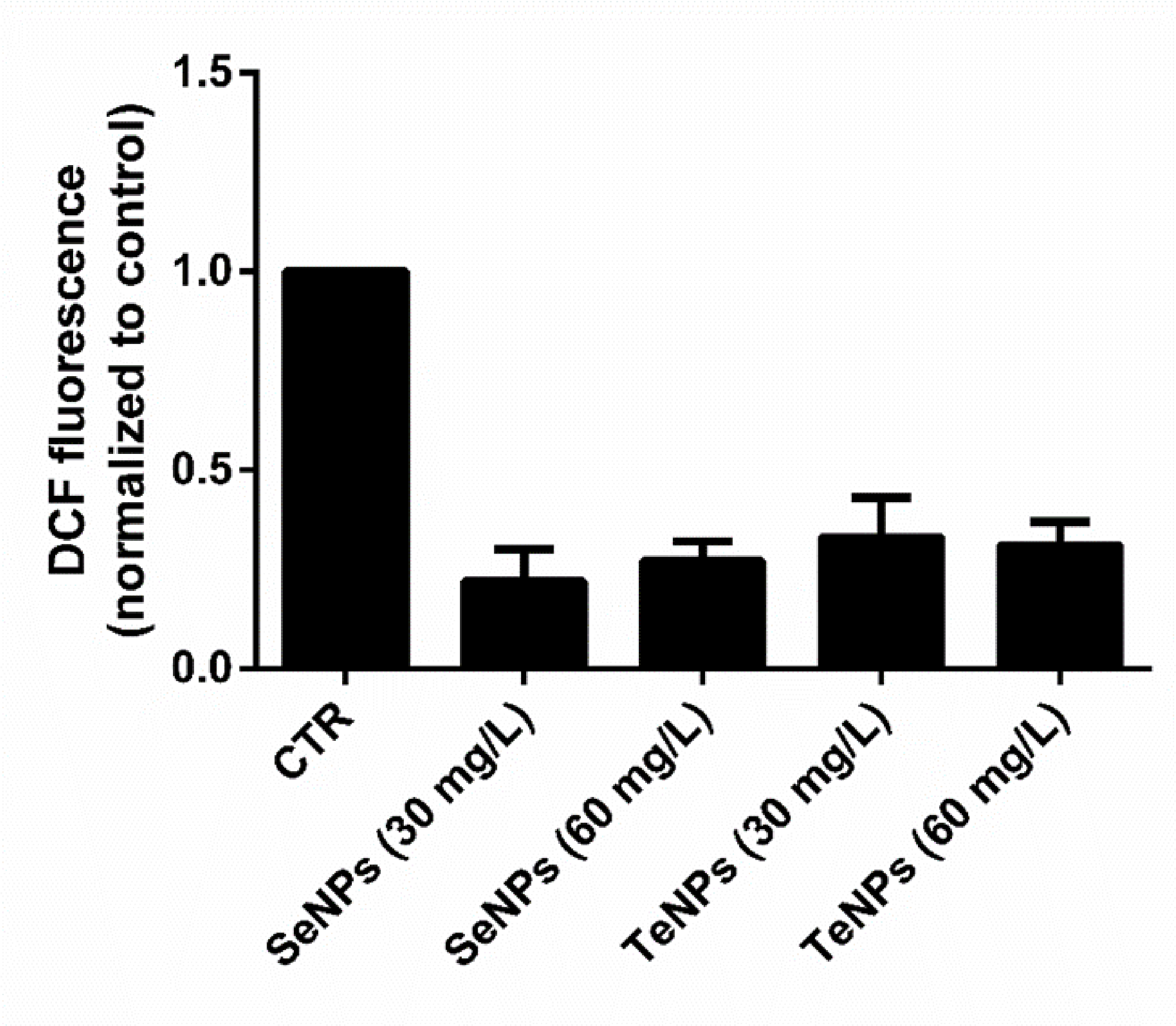


**Figure S1:** Evaluation of the interaction between Se and Te NPs with DCF reagent. CTR is microbial cells not exposed to nanoparticles.
